# Bilateral Long-Lasting Changes in Multimodal Sensitivity After Cutaneous High-Frequency Electrical Stimulation: A New Comprehensive Dermatomal Examination

**DOI:** 10.3390/jcm15197425

**Published:** 2026-09-24

**Authors:** Isabel Escobar-Sánchez, Julian Taylor, Juan José Fernández-Pérez, Marta Ríos-León

**Affiliations:** 1 Sensorimotor Function Group, Hospital Nacional de Parapléjicos (SESCAM), 45071 Toledo, Spain; isaesanchez2@gmail.com (I.E.-S.); juliantaylorgreen2@gmail.com (J.T.); 2 Harris Manchester College, University of Oxford, Oxford OX1 3TD, UK; 3 Instituto de Investigación Sanitaria de Castilla-La Mancha (IDISCAM), 45071 Toledo, Spain; 4 Grupo de Investigación de Neurociencias Aplicadas a la Rehabilitación (GINARE), Clínica Neuron Rehabilitación, 28293 Alcorcón, Spain; jjferpe55@gmail.com; 5 Department of Basic Medical Sciences, School of Medicine, San Pablo-CEU University, 28660 Boadilla del Monte, Spain

**Keywords:** long-term potentiation, high-frequency stimulation, multimodal sensitivity, nociception, pain

## Abstract

**Background/Objectives**: Little is known about long-term changes in sensitivity and pain following cutaneous 100 Hz high-frequency stimulation (HFS) in humans. Thus, the aim of this study was to investigate long-lasting changes in multimodal sensitivity after HFS using a comprehensive systematic dermatomal examination, including two innovative protocols, and to identify common factors associated with these changes. **Methods**: Long-lasting changes were assessed with thermal and mechanical bedside and quantitative sensory testing at the homotopic (ipsilateral C6 or L4 dermatomes), adjacent heterotopic (ipsilateral C5 or L3 dermatomes) and contralateral dermatomal sites (C6 or L4 dermatomes) in two sessions (session A: upper limb dermatomal examination protocol; session B: lower limb dermatomal examination protocol). Before and after 100 Hz HFS, test stimuli (TS) were administered to the ipsilateral C6 dermatome for session A or ipsilateral L4 dermatome for session B. Individuals reported pain intensity or sensation related to TS, and fulfilled questionnaires associated with psychological health, pain descriptors, or perceived quality of life. **Results**: Long-lasting changes in both cold and mechanical sensitivity were observed up to 30 min following HFS at all sites in both sessions (*p* < 0.05). No significant differences between sessions were found for significant HFS-induced changes in sensitivity (*p* > 0.05). Common changes in cold sensitivity were dependent on age (*p* < 0.05). Fatigue was associated with common changes in mechanical or cold sensitivity (*p* < 0.05). **Conclusions**: Bilateral long-lasting changes in multimodal sensitivity after HFS have been revealed in a novel comprehensive systematic dermatomal examination. These findings provide new insight into persistent pain mechanisms and an innovative approach for its management.

## 1. Introduction

Cutaneous electrical high-frequency stimulation (HFS) induces long-term potentiation (LTP) [1]. LTP is considered a neural mechanism of synaptic plasticity involving a long-lasting increase in synaptic efficacy within spinal nociceptive circuits [2]. Indeed, HFS-induced LTP contributes to long-lasting experimental hyperalgesia and allodynia following sensitization of spinal nociceptive pathways [1,3,4,5,6,7]. As HFS-induced LTP provides mechanistic insight into synaptic plasticity [2], understanding HFS-induced plasticity (LTP) may provide information related to neural sensitization and pain amplification [2], which are associated with persistent pain. Furthermore, research focused on LTP for individuals with defined central or peripheral nervous system injury might lead to an appropriate understanding of the pathophysiology and time course of changes in nociception, which may promote the development of novel approach for the management of pain conditions [1,4,5]. In fact, in individuals with unilateral neuropathic pain, widespread changes in nociception and pain, including adjacent and contralateral testing sites, have been recently revealed [8,9]. Thus, consistent information extracted from consistent and reproducible comprehensive dermatomal assessment protocols is now needed to document rapid HFS-induced changes in multimodal sensitivity related to nociception and experimental pain.

Cutaneous electrical HFS has previously been shown to induce LTP-like pain amplification (pain-LTP) via activation of C-fiber nociceptors [3,5,10]. Indeed, a small-diameter electrode may facilitate the activation of superficial nociceptive Aδ- and C-fibers [3]. Electrical stimuli applied within the HFS area lead to an increase in pain intensity at the HFS site (homotopic hyperalgesia (primary hyperalgesia)) [2,3,11]. Furthermore, changes in pain sensitivity, particularly changes in mechanical pain sensitivity (pinprick), at adjacent area to HFS site (heterotopic hyperalgesia) have also been observed [1,2,3,4,12,13,14,15]. Additionally, a long-lasting increase in pain sensations (heterotopic) evoked by dynamic mechanical tactile [3,14,16] or thermal stimuli [1,17] were also revealed. Nevertheless, findings related to thermal sensitivity after HFS, particularly cold stimuli, were unclear [15]. These findings may suggest that LTP-like homosynaptic and/or heterosynaptic mechanisms might be associated with the development of primary and secondary hyperalgesia [2,11,18,19,20], which might lead to central and peripheral sensitization to multimodal stimuli at the adjacent, same, or remote dermatomes. Lastly, psychological or demographic factors, which may modulate pain perception, might also influence HFS-induced pain-LTP changes [1,14,21,22].

Most studies performed upper limb examination for research in LTP [1,3,4,5,6,10,11,13,14,15,16,23,24,25]. Nevertheless, no systematic HFS-induced pain-LTP experimental protocol applied to lower limbs dermatomes has been previously developed. Likewise, no comparison between different systematic dermatomal examination protocols has been previously investigated to reproduce findings and to promote new comprehensive assessment tools for studying chronic pain conditions. Additionally, little is known about demographic or clinical factors related to pain-LTP.

This study aimed to investigate long-lasting changes in mechanical or thermal TS at heterotopic and homotopic dermatomal sites after cutaneous electrical HFS applied at upper or lower limbs, to compare systematic upper and lower limbs dermatomal examination protocols, and to identify the impact of clinical and demographic factors on induced long-lasting changes.

## 2. Materials and Methods

This study was performed in accordance with the Strengthening the Reporting of Observational Studies in Epidemiology (STROBE) guidelines and the Declaration of Helsinki [26,27]. The study protocol was approved by the local Clinical Research Ethics Committee (Reference No. 2559/674).

### 2.1. Subjects

The recruitment was performed between September 2023 and December 2024 at a national reference hospital in Toledo, Spain. Prior to the inclusion in the study, all individuals provided and signed written informed consent.

A total of 33 healthy individuals aged 18–70 years were included (51.5% women, mean age = 29.2 ± 8.1; Table 1). History of chronic pain and/or psychiatric or neurological diseases [1], neuropathy or peripheral nervous system injury [1], diseases causing potential neural damage (e.g., oncological diseases, diseases of the immune system, diabetes) [1], previous spine surgery, a history of upper- or lower-limb surgery or injury, altered sensitivity in TS areas [1], orofacial pain, or frequent headaches were the exclusion criteria.

### 2.2. Experimental Protocol

The present protocol was developed from findings obtained in our previous upper-limb dermatomal study [1].

Participants completed two testing sessions in a randomized order, each lasting approximately 1 h. Sensory testing was conducted in a quiet laboratory environment maintained at 22–26 °C. While seated comfortably, participants received detailed instructions regarding the experimental procedure [1]. Individuals were instructed to avoid caffeine intake and strenuous physical activity during the 24 h preceding the experiment [1].

Session A (upper limb protocol, Figure 1) consisted of baseline testing with TS (*see below*) administered to the C5 and C6 dermatomes (dominant side), and contralateral C6 dermatome [1] at 5 min intervals for 15 min, including an additional assessment 25 min before high-frequency stimulation (HFS). After baseline testing, cutaneous electrical HFS (conditioning stimuli, CS) was delivered to the C6 dermatome (dominant side) for 60 s (*see HFS section below*) [1]. An 11-point numerical pain rating scale (NRS; 0 (no pain)–10 (maximum pain)) was used to determine pain intensity during the electrical stimulus [28]. Following HFS, TS were administered to the ipsilateral C5 and C6 dermatomes (dominant side), and contralateral C6 dermatome to assess heterotopic and homotopic effects at 5 min intervals [1] for 30 min.

Session B (lower limb protocol, Figure 1) consisted of baseline testing with TS (*see below*) administered to the L3 and L4 dermatomes (dominant side), and contralateral L4 dermatomes according to the assessment schedule used in session A. After baseline testing, HFS (CS) was delivered to the L4 dermatome (dominant side) for 60 s (*see below*). Pain intensity during electrical stimulus was recorded (NRS, 0–10) [28]. Following HFS, TS were administered to the ipsilateral L3 and L4 dermatomes (dominant side), and contralateral L4 dermatome at the same time points described for session A.

A one-week interval separated the two sessions. The study compared post-HFS effects on pain and sensory intensity with the 25 min baseline measurements recorded from each individual in both sessions. The study hypothesis was not disclosed to participants.

#### 2.2.1. High-Frequency Stimulation (HFS)

Electrical stimulation was delivered through a bipolar concentric electrode (522100, BCS-probe 90 mm straight, Inomed Medizintechnik GmbH, Emmendingen, Germany; diameter, 2 mm) connected to a constant current stimulator (DS7A, Digitimer Ltd., Welwyn Garden City, Hertfordshire, UK) [1,3,4]. In session A, the electrode was positioned on the dominant forearm, 5 cm distal to the cubital fossa (American Spinal Injury Association Impairment Scale (AIS) Key Sensory Point [29]), corresponding to the C6 dermatome [1,3,4]. In session B, stimulation was administered over the medial malleolus of the dominant ankle, corresponding to the L4 dermatome (AIS Key Sensory Point [29]).

Due to its small diameter, the electrode generated a relatively high current density at low stimulation intensities, thereby facilitating the recruitment of superficial nociceptive Aδ- and C-fiber afferents [1,3]. For each participant, the stimulation intensity was individually set to produce a pain rating of 4/10 (“Pain-4”) on the NRS, where 0 represented no pain and 10 the worst pain possible [1,28]. Before electrical CS, “Pain-4” was determined using single electrical pulses (100 Hz, 1 ms pulse width) [1]. The intensity for electrical detection threshold, electrical pain threshold, and “Pain-4” were determined by increasing the current in 0.1 mA steps (single electrical pulses). Participants indicated the point at which they first perceived the electrical stimulus, experienced pain, or rated the pain as 4/10, respectively [1]. The “Pain-4” intensity was calculated as ten times the electrical detection threshold. Each individual was acquainted with the electrical stimuli before assessment [1].

HFS (CS) consisted of trains lasting 1 s, each containing five pulses at 100 Hz. The trains were delivered every 10 s for one minute to induce pain-LTP [1,4,15]. Pain intensity during HFS was recorded every 10 s using the NRS, ranging from 0 (no pain) to 10 (maximum pain) [1].

If the stimulation produced intolerable pain (NRS), participants were permitted to withdraw the stimulated arm or ankle from the electrode at any point during the procedure [1].

#### 2.2.2. Test Stimuli (TS)

Two types of test stimuli (TS), thermal and mechanical, were used to detect changes in sensory and pain sensitivity.

In session A, TS were administered to the forearm, 5 cm distal to the cubital fossa, corresponding to the C6 dermatome, on both dominant (homotopic site) and nondominant sides (contralateral C6 dermatome, control site) [1]. In session B, TS were applied to the medial malleolus, corresponding to the L4 dermatome, on the dominant (homotopic site) and nondominant sides (contralateral L4 dermatome, control site).

For session A, TS were also administered to the lateral (radial) aspect of the antecubital fossa, just proximal to the elbow, corresponding to the C5 dermatome (heterotopic site) [1], according to the AIS Key Sensory Point [29], on the dominant side. For session B, TS were also applied to the medial femoral condyle above the knee corresponding to the L3 dermatome (heterotopic site), according to the AIS Key Sensory Point [29], on the dominant side.

The selected TS sites were included to assess local spinal modulatory effects after HFS [1].

##### Mechanical TS

Mechanical TS were administered using a brush (SenseLab 05, Somedic AB, Sösdala, Sweden), von Frey filament (Stoelting Co., Wood Dale, IL, USA; No. 5.88, nominal buckling force 588.2 mN), and cotton bud (Q-tip, Evermark, LLC, Monsey, NY, USA) at the test sites to assess dynamic mechanical allodynia (brushing), mechanical pain sensitivity (pinprick), and dynamic mechanical detection sensitivity (light touch sensation), respectively [1]. The sensation evoked by each stimulus at the selected test area was evaluated against the V2 dermatome (AIS Key Sensory Point, zygomatic bone [29]) as an additional control site [1]. The cotton bud and brush were administered using 1 and 4 strokes (1 to 2 cm length), respectively [1]. The von Frey filament was applied perpendicularly to the skin surface (test site) for up to 2 s until bending of the filament occurred. It was maintained in position for an additional 2 s, and subsequently removed [1]. These mechanical TS predominantly activate Aβ-fiber low-threshold mechanoreceptors and Aδ-fiber nociceptors [15].

For these mechanical TS (brush, cotton bud, von Frey Filament), participants rated the perceived intensity of TS at the test areas relative to the V2 dermatome on a 3-point scale (1: less intense than V2, 2: equal to V2, 3: greater than V2) [1]. This instrument was based on validated tools commonly applied in standardized sensory examinations [1,30,31]. Furthermore, pain intensity related to TS was determined via NRS (0–10) [1,28]. Assessments of sensation and pain intensity associated with TS were conducted every 5 min before HFS and continued for 30 min post-HFS (i.e., 5, 10, and 15 min prior to HFS, and 5, 10, 15, 20, 25, and 30 min after HFS).

Additionally, pressure pain sensitivity was evaluated using the pressure pain threshold (PPT), defined as the minimum pressure required for a pressure sensation to be perceived as painful [32]. PPTs were assessed bilaterally by an experienced evaluator using an electronic algometer (FPIXTM, Wagner Instruments, Greenwich, CT, USA) at the test sites in a random order, 25 min prior to HFS and 30 min after HFS. Regarding test sites for PPTs, C5 dermatome was assessed as described above (*see Test stimuli (TS)*); however, C6 and contralateral C6 dermatomes were assessed over second metacarpal space at the dominant and nondominant hands (homotopic and control sites, respectively). Pressure was applied to each test point at around 30 kPa/s. The mean of 3 measurements obtained at each site was included in the primary analysis. To minimize potential temporal pain summation effects, consecutive measurements were separated by a 30 s interval [33]. As a quantitative sensory testing (QST) measure, algometry has demonstrated excellent consistency between examiners, with an intraclass correlation coefficient of 0.91 (95% confidence interval (CI): 0.82–0.97) [34].

##### Thermal TS

Cold and heat sensitivity were assessed with thermal rollers (Somedic SenseLab AB, Sösdala, Sweden; Roll-Temp II) at the test areas, with each stimulus applied over a 5 cm long stroke. Thermal stimulation was administered using rollers maintained at 25 °C (cold) and 40 °C (heat). These thermal TS predominantly activate Aδ- and C-fiber afferents [15].

For these thermal TS (thermal rollers), participants rated the perceived intensity of TS at the test areas relative to the V2 dermatome on a 3-point scale (1: less intense than V2, 2: equal to V2, 3: greater than V2) [1]. This instrument was based on validated tools commonly applied in standardized sensory examinations [1,30,31]. Furthermore, the pain intensity related to TS was determined via NRS (0–10) [1,28]. Sensation and pain intensity associated with TS were evaluated before and up to 30 min after HFS (5 min intervals). Thus, these assessments were performed at 25 min before HFS, and 5, 10, 15, 20, 25, and 30 min after HFS.

Additionally, thermal TS also included thermal QST. Thermal detection thresholds (heat, cold) and thermal pain thresholds (cold, heat) were assessed. Testing was performed at the test sites using a Thermal Cutaneous Stimulator 2.2 (TCS 2.2, QST.Lab, Strasbourg, France). The device is a contact thermode system capable of producing temperature variations of up to 300 °C/s [35]. It comprises a control unit coupled to a thermode that operates using thermoelectric Peltier technology [35]. Thermal threshold testing was performed with the method of limits [35,36]. A handheld 6.6 × 4.0 cm contact thermode (stimulation surface: 9 cm^2^) with baseline 32 °C and 1 °C/s upslope was used. Cut-off temperatures were 50 °C and 0 °C [36]. Baseline thermal thresholds (heat/cold) were obtained from the average of three consecutive measurements at each test location. Thermal thresholds were assessed before and up to 30 min after HFS as follows: cold detection and cold pain thresholds were assessed 5,10, and 15 min before HFS and 15 and 30 min after HFS; heat detection and heat pain thresholds were assessed 25 min prior to HFS and 30 min after HFS.

### 2.3. Neuropathic Sensations

HFS-induced neuropathic sensory characteristics were evaluated using 7 items of the DN4 questionnaire: electric shocks, burning, painful cold, pins and needles, tingling, itching, and numbness [37]. The participants reported the intensity of each neuropathic sensation using NRS (0: absence; 10: maximum intensity) before (5, 10, and 15 min prior to HFS) and up to 30 min after HFS (5, 10, 15, 20, 25, and 30 min after HFS).

### 2.4. Psychological Health and Perceived Quality of Life

The Pain Anxiety Symptoms Scale-20 (PASS-20) is a 20-item self-report tool that measures 4 components of anxiety associated with pain (i.e., cognitive responses associated with anxiety, fearful thinking, escape and avoidance, physiological anxiety responses). Items are scored on a six-point Likert scale, where 0 represented never and 5 represented always. The total score (0–100 points) indicates general level of pain-related anxiety [38,39,40].

The paper-based five-level version of EuroQol-5D (EQ-5D-5L) was used to determine health-related quality of life. This tool assesses self-care, mobility, depression/anxiety, independent daily activities, and pain domains. Each domain was scored from 5 (extreme problems) to 1 (absence of problems). All responses were converted into an index number, which represents the health state from 1 (optimal health) to 0 (health state equivalent to death) [41].

The Patient-Reported Outcomes Measurement Information System 29-item (PROMIS-29) profile version 2.1 (PROMIS-29 Profile v2.1) was also used. This tool is a 29-item measure that assesses seven health-related quality of life domains (physical function, depression, anxiety, sleep disturbance, fatigue, social participation and pain interference), in addition to one item to assess pain intensity [42]. Each domain, except for pain intensity (NRS, 0–10), consists of 4 items scoring on a 5-point Likert scale (1–4). The raw scores for each domain were summed separately (1–20) and converted using the T-score metric system. Raw scores were converted into standardized T-scores with a mean of 50 and a standard deviation (SD) of 10 based on the US reference population. Higher PROMIS function T-scores indicate better functioning, whereas higher symptom T-scores are associated with worse symptom burden [42].

### 2.5. Statistical Analysis

Data analysis was conducted using GraphPad Prism version 11.0.2 (GraphPad Software, San Diego, CA, USA) and SPSS version 22.0 (IBM Corp., Armonk, NY, USA). Results are expressed as mean, standard deviation (SD), standard error (SE), or 95% CI. The Shapiro–Wilk test revealed that data did not follow a normal distribution (*p* < 0.05); therefore, nonparametric tests were used. The Friedman test followed by post-hoc pairwise tests using the Wilcoxon matched-pairs signed-rank test were performed to investigate change in TS sensation, pain intensity following HFS, or pain intensity during HFS (trend of pain across time). Overall differences in TS sensations or pain intensity, and neuropathic sensations before and after HFS were calculated with the Wilcoxon signed-rank test. Differences in HFS-induced changes in sensitivity (quantified as the difference between post- and pre-HFS TS values (i.e., difference between TS values after and before HFS), TS2-TS1) between protocols (sessions) were analyzed using the Mann–Whitney U test. Additionally, the possible influence of baseline clinical and demographic characteristics on HFS-induced changes in sensation or pain intensity were analyzed with the χ2 tests of independence, the Mann–Whitney U test, or Kruskal–Wallis test followed by post-hoc pairwise tests to control for multiple comparisons. Finally, Spearman’s rho (r_s_) and locally estimated scatterplot smoothing (LOESS) regression analysis were used to analyze the association between clinical variables and changes in sensitivity, in addition to correlation between both protocols (sessions). Correlation analysis was also corroborated by linear regression and/or polynomial regression coefficients analysis considering LOESS plot. An additional preliminary intraclass correlation coefficient (ICC) analysis was also performed to support the significant correlation and regression analyses between protocols (sessions), thereby exploring potential estimates of preliminary reliability (consistency) of these protocols. The statistical analysis was conducted at a 95% confidence level. A *p* value <0.05 was considered statistically significant.

## 3. Results

### 3.1. Clinical and Demographic Characteristics

Thirty-three healthy participants (51.5% women, mean age = 29.2 (SD 8.1)) were included. Demographic and clinical data are shown in Table 1. No significant differences in baseline characteristics were reported for sex or body mass index (BMI), except for age: younger people showed higher scores in social participation domain (PROMIS-29 Profile v2.1) (*p* = 0.026).

### 3.2. Perception of CS

After HFS, no visible skin injuries occurred. Skin blushing under the stimulated area was presented. In both sessions, a significant increase in pain intensity was revealed during the 60 s (Figure 2; *p* < 0.001).

No significant differences between protocols (sessions) were observed (*p* > 0.05).

### 3.3. Effect of Electrical HFS on Induced Neuropathic Sensations

In session A, during the 30 min after HFS, a decrease in number of induced neuropathic sensations was observed (5 min: 5; 30 min: 2) and a significant decrease in intensity of induced neuropathic sensations was reported (5 min: 1.2 (SE 0.32); 30 min: 0.06 (SE 0.06); *p* = 0.02). The most common neuropathic sensations after HFS were pins and needles, itching or burning sensations.

In session B, a decrease in number of induced neuropathic sensations was observed (5 min: 4; 30 min: 1) and a significant decrease in intensity of induced neuropathic sensations was reported (5 min: 1.1 (SE 0.3); 30 min: 0.06 (SE 0.06); *p* = 0.002). The most common neuropathic sensations after HFS were pins and needles, tingling or burning sensations.

No significant differences between protocols (sessions) were found (*p* > 0.05).

### 3.4. Changes in Mechanical and Thermal Sensitivity After HFS

#### 3.4.1. Session A (Upper Limb)

Significant changes before (pre-HFS) and after HFS (post-HFS) in dynamic mechanical detection sensitivity (cotton bud) were found: an increase in dynamic mechanical detection sensitivity was found at ipsilateral C6 dermatome at 5 min (*p* = 0.011), 25 min (*p* = 0.014) and 30 min (*p* = 0.022) after HFS.

Significant changes pre- and post-HFS in mechanical pain sensitivity (PPTs) at all measured points were found (Figure 3; *p* < 0.02): a significant decrease in PPTs over all measured points (C6: *p* = 0.006; C5: *p* = 0.014; contralateral C6: *p* = 0.007) were revealed. Greater changes related to the increase in mechanical pain hypersensitivity (PPTs) were found in contralateral C6 dermatome, followed by ipsilateral C6 and C5 dermatomes (Table 2). Additionally, significant changes in mechanical pain sensitivity (pinprick) were also found: mechanical hypersensitivity (pinprick) after HFS was revealed at ipsilateral C6 dermatome (10 min: *p* = 0.049; 15 min: *p* = 0.015; 20 min: *p* = 0.001; 25 min: *p* = 0.021; 30 min: *p* = 0.034) and contralateral C6 dermatome (10 min: *p* = 0.006; 25 min = 0.009).

Significant changes pre- and post-HFS in cold sensitivity (QST) at ipsilateral C5 and C6 dermatomes and contralateral C6 dermatome at 15 min after HFS (C5: *p* = 0.002; C6: *p* < 0.001; contralateral C6: *p* < 0.001) and 30 min after HFS (C5: *p* = 0.001; C6: *p* < 0.001; contralateral C6: *p* = 0.028) were found (Figure 4). Greater changes related to the increase in cold hypersensitivity were found in ipsilateral C6 dermatome at 15 min after HFS (2.2 (SE 0.3)) and at 30 min after HFS (Table 2), followed by ipsilateral C5 dermatome (15 min after HFS: 1.0 (SE 0.5); 30 min after HFS: Table 2) and contralateral C6 dermatome (15 min after HFS: 0.8 (SE 0.2); 30 min after HFS: Table 2).

**Table 2:** Changes in mechanical and thermal sensitivity, assessed with quantitative sensory testing (QST), pre- and post-HFS (30 min) in each session (n = 33).

Type of Sensitivity	Changes in Sensitivity (Session A)	Changes in Sensitivity (Session B)
**Mechanical sensitivity** (PPTs, kPa)	C6 dermatome: −57.0 (SE 19.0)^δ^ C5 dermatome: −45.0 (SE 19.0)^δ^ Contralateral C6 dermatome: −61.0 (SE 19.0)^δ^	L4 dermatome: −76.0 (SE 24.0)^δ^ L3 dermatome: −50.0 (SE 18.0)^δ^ Contralateral L4 dermatome: −51.0 (SE 15.0)^δ^
**Thermal sensitivity**		
Cold sensitivity (cold detection threshold, °C)	C6 dermatome: 1.7 (SE 0.3)^δ^ C5 dermatome: 1.2 (SE 0.4)^δ^ Contralateral C6 dermatome: 0.5 (SE 0.2)^δ^	L4 dermatome: 1.2 (SE 0.4)^δ^ L3 dermatome: 0.9 (SE 0.4)^δ^ Contralateral L4 dermatome: 1.1 (SE 0.5)^δ^
Heat sensitivity (heat detection threshold, °C)	C6 dermatome: −0.1 (SE 0.2) C5 dermatome: −0.7 (SE 0.4)* Contralateral C6 dermatome: −0.2 (SE 0.2)	L4 dermatome: 0.1 (SE 0.6) L3 dermatome: 1.1 (SE 0.3)^δ^ * Contralateral L4 dermatome: −0.6 (SE 0.5)

Data are presented as means (standard error (SE)). Abbreviations: HFS: high-frequency stimulation; PPTs: Pressure Pain Thresholds. ^δ^ Intra-group comparisons: significant changes in sensitivity pre- and post-HFS (30 min) for each session (*p* < 0.05). * Inter-group comparisons: significant differences between session A (upper limb protocol, HFS at C6 dermatome) and session B (lower limb protocol, HFS at L4 dermatome) (*p* < 0.05). *It is important to note that no significant changes pre- and post-HFS in heat sensitivity were found in ipsilateral C5 dermatome, unlike ipsilateral L3 dermatome, suggesting no clear trend in heat sensitivity after HFS in session A, no clear overall trend in heat sensitivity in both sessions and no clear difference between them.*

No other significant changes pre- and post-HFS were found (*p* > 0.05).

#### 3.4.2. Session B (Lower Limb)

Significant changes pre- and post-HFS in dynamic mechanical allodynia (brushing) were found: an increase in dynamic mechanical allodynia were found at ipsilateral L4 dermatome at 5 min after HFS (*p* = 0.034).

Significant changes pre- and post-HFS in mechanical pain sensitivity at all measured points were found (Figure 5; *p* < 0.02): a significant decrease in PPTs over all measured points (L4: *p* = 0.001; L3: *p* = 0.013; contralateral L4: *p* = 0.003) were revealed. Greater changes related to the increase in mechanical pain hypersensitivity were found in ipsilateral L4 dermatome, followed by contralateral L4 dermatome and ipsilateral L3 dermatome (Table 2). Additionally, significant changes in mechanical pain sensitivity (pinprick) were also found: mechanical hypersensitivity (pinprick) after HFS was revealed at ipsilateral L4 dermatome (5 min: *p* = 0.006; 10 min: *p* = 0.005; 15 min: *p* = 0.007; 20 min: *p* = 0.007; 25 min: *p* = 0.006; 30 min: *p* = 0.031).

Significant changes pre- and post-HFS in heat sensitivity (QST) at ipsilateral L3 dermatome were found (Table 2, *p* = 0.02). Significant changes pre- and post-HFS in cold sensitivity (QST) at ipsilateral L4 and L3 dermatomes at 15 min after HFS (L4: *p* < 0.001; L3: *p* = 0.001), and at ipsilateral L4 and L3 dermatomes and contralateral L4 dermatome at 30 min after HFS (L4: *p* = 0.003; L3 *p* = 0.029; contralateral L4: *p* < 0.001) were revealed (Figure 6). Greater changes related to the increase in cold hypersensitivity were found in ipsilateral L4 dermatome at 15 min after HFS (1.5 (SE 0.5)) and at 30 min after HFS (Table 2), followed by contralateral L4 dermatome (30 min after HFS: Table 2) and ipsilateral L3 dermatome (15 min after HFS: 1.3 (SE 0.3); 30 min after HFS: Table 2).

No other significant changes pre- and post-HFS were found (*p* > 0.05).

#### 3.4.3. Changes Pre- and Post-HFS in Both Sessions

Significant differences between protocols (sessions) were revealed in changes pre- and post-HFS in heat sensitivity (QST) at the ipsilateral C5 and L3 dermatomes (*p* < 0.001; Table 2). No other significant differences were found in changes pre- and post-HFS in mechanical or thermal sensitivity with similar tendencies in both protocols (Table 2).

Significant associations between changes pre- and post-HSF in mechanical or cold sensitivity in session A and those in session B were also found (PPTs in contralateral C6 and L4 dermatomes: r_s_ = 0.392, *p* = 0.024; cold sensitivity (QST) in ipsilateral C6 and L4 dermatomes: r_s_ = 0.551, *p* = 0.001; cold sensitivity (QST) in ipsilateral C5 and L3 dermatomes: r_s_ = 0.357, *p* = 0.041; cold sensitivity (QST) in contralateral C6 and L4 dermatomes: r_s_ = 0.465, *p* = 0.006; Table S1).

### 3.5. Influence of Clinical and Demographic Characteristics on Changes in Mechanical and Thermal Sensitivity Related to HFS

#### 3.5.1. Session A

In upper limbs protocol (session A), significant age differences in mechanical pain sensitivity (pinprick, *p* < 0.001) at ipsilateral C6 dermatome at 30 min after HFS were found: older participants exhibited mechanical pain hypersensitivity more frequently than younger individuals (<26 years: 10%; >26 years: 44.4%). Additionally, significant ages differences in changes pre- and post-HFS (30 min) for cold sensitivity (QST) at ipsilateral and contralateral C6 dermatomes were also revealed (*p* = 0.025): younger participants showed greater changes in cold sensitivity than older individuals (<26 years: 1.1 (SE 0.6); >26 years: −0.1 (SE 0.3)), indicating a trend toward increased cold sensitivity in younger participants.

Significant BMI differences in cold sensitivity (QST, *p* = 0.049) at ipsilateral C6 dermatome at 15 min after HFS were revealed: higher changes in cold hypersensitivity were found in individuals with higher BMI, particularly in participants with obesity (BMI ≥ 30; cold sensitivity: 2.2 (SE 0.7)) compared with those with normal weight (BMI: 18.5–24.9; cold sensitivity: 2.1 (SE 0.4)).

No significant sex differences were found (*p* > 0.05).

Additionally, changes in cold sensitivity (QST) at ipsilateral C6 dermatome were associated with fatigue (PROMIS-29 Profile v2.1) (r_s_ = 0.422, *p* = 0.045; Table S2): the greater fatigue, the higher cold hypersensitivity. Changes in cold sensitivity (QST) at ipsilateral C5 dermatome were also associated with sleep disturbance (PROMIS-29 Profile v2.1) (r_s_ = 0.535, *p* = 0.008; Table S2): greater cold hypersensitivity was associated with a trend toward higher levels of sleep disturbance.

#### 3.5.2. Session B

In lower limbs protocol (session B), significant age differences in changes pre- and post-HFS (30 min) for mechanical pain sensitivity (PPTs, *p* = 0.02) at ipsilateral L4 dermatome, and cold sensitivity (QST, *p* = 0.07) at ipsilateral L3 dermatome were revealed: younger participants showed greater changes in mechanical pain sensitivity than older (<26 years: −9.0 (SE 4.4); >30 years: −2.8 (SE 3.5)), in addition to greater changes in cold sensitivity (26–30 years: 0.3 (SE 0.7); >30 years: −0.02 (SE 1.3)).

Significant BMI differences in changes pre- and post-HFS (30 min) for mechanical pain sensitivity (PPTs, *p* = 0.015) at ipsilateral L4 dermatome and heat pain sensitivity (QST, *p* = 0.003) at ipsilateral L3 dermatome were also found: higher changes in mechanical pain sensitivity and heat pain sensitivity were found in individuals with higher BMI, particularly in participants with obesity (BMI ≥ 30; PPTs: −12.2 (SE 0.7); heat pain sensitivity: 2.3 (SE 1.6)) compared with those with normal weight (BMI: 18.5–24.9; PPTs: −6.5 (SE 2.3); heat pain sensitivity: 0.1 (SE 0.4)).

Significant sex differences in changes pre- and post-HFS (15 min) for cold sensitivity (QST) at the contralateral L4 dermatome were also found: women showed greater changes in cold sensitivity compared with men (0.7 (SD 0.6) vs. −1.3 (SE 0.9), *p* = 0.045), suggesting a trend toward increased cold sensitivity in women but not in men.

Additionally, changes in mechanical pain sensitivity (PPTs) at contralateral L4 dermatome were associated with physical function, anxiety, fatigue, and sleep disturbance (PROMIS-29 Profile v2.1) (r_s_ = −0.384, *p* = 0.027; r_s_ = −0.395, *p* = 0.023; r_s_ = −0.443, *p* = 0.01; r_s_ = −0.502, *p* = 0.003; respectively): higher levels of anxiety, lower physical functioning or a trend toward greater fatigue or sleep disturbance were associated with increased mechanical pain hypersensitivity (Table S2). Changes in cold sensitivity (QST) at ipsilateral L3 dermatome were also associated with quality of life (r_s_ = −0.377, *p* = 0.031; Table S2): greater cold hypersensitivity was associated with a trend toward reduced quality of life.

### 3.6. Influence of Evoked Pain During HFS on Changes in Mechanical and Thermal Sensitivity After HFS

In session A, the pain intensity during the first 10 s interval of HFS was associated with changes in PPTs at ipsilateral C6 dermatome (r_s_ = −0.387, *p* = 0.026; Table S2). Pain intensity during the first 30 s interval of HFS was also associated with changes in cold sensitivity (QST) at ipsilateral C6 dermatome (r_s_ = 0.422, *p* = 0.015; Table S2), ipsilateral C5 dermatome (r_s_ = 0.456, *p* = 0.008; Table S2) and contralateral C6 dermatome at 30 min after HFS (r_s_ = 0.408, *p* = 0.018; Table S2). The higher pain intensity, the greater cold or mechanical hypersensitivity.

In session B, temporal summation of pain during HFS was associated with changes in cold sensitivity (QST) at contralateral L4 dermatome at 15 min after HFS (r_s_ = 0.467, *p* = 0.006; Table S2). Additionally, the pain intensity during the first 40 s interval of HFS was associated with changes in cold sensitivity (QST) at ipsilateral L4 dermatome at 15 min after HFS (r_s_ = 0.388, *p* = 0.026; Table S2). The higher pain intensity, the greater cold hypersensitivity.

## 4. Discussion

For the first time, significant and simultaneous long-lasting changes in mechanical pain sensitivity and cold sensitivity at the conditioned area, adjacent test and contralateral test sites following HFS applied to both upper limb and lower limb dermatomal examinations were revealed. In fact, this is the first study investigating long-lasting changes in different modalities of sensitivity after electrical HFS applied to lower limbs and comparing two protocols related to LTP-like mechanisms after HFS applied at different locations (i.e., common upper limb locations and new lower limb locations). No significant differences were found between protocols (sessions) in significant changes in sensitivity related to HFS in both sessions, with similar tendencies observed in both protocols (sessions). In addition, a clear and significant overall trend toward mechanical hypersensitivity at contralateral sites (control sites) and cold hypersensitivity at all sites were revealed between both protocols (sessions), suggesting the potential application of each protocol for studying LTP-like mechanisms related to different chronic pain conditions. Similar HFS-induced consistent neuropathic sensations were also evoked in both protocols (sessions). The results also suggest a common effect of age, together with a potential influence of clinical factors such as fatigue, on the HFS-induced long-lasting changes in thermal or mechanical sensitivity across both sessions in healthy individuals.

### 4.1. HFS Induces Consistent Neuropathic Sensations and Bilateral Long-Lasting Mechanical Hyperalgesia

According to previous studies [1,24], electrical HFS evoked the perception of neuropathic sensations (e.g., pins and needles or burning sensations), which might be associated with the activation of C- and Aδ-fibers [1,24]. Furthermore, the most common neuropathic sensations evoked were similar between protocols (sessions), which might suggest a potential role of LTP-like mechanisms in neuropathic sensations related to pain.

Furthermore, this study reveals, for the first time, bilateral long-lasting mechanical pain hypersensitivity following HFS applied at different locations (protocols). Previous studies revealed primary and/or secondary mechanical hyperalgesia at the adjacent conditioned site area after HFS applied to upper limb dermatomes [1,3,4,5,10,11,14,25,43,44]. Furthermore, long-lasting mechanical hypersensitivity to pinprick stimuli has been previously reported [1,5,23,43,44], including an increase in pinprick-evoked brain potentials [45]. Nevertheless, this is the first study revealing the presence of both primary hyperalgesia and secondary mechanical hyperalgesia, measured via PPTs, at the adjacent conditioned site and remote sites after HFS in different locations (protocols), possibly mediated by primary nociceptive C-fibers as “facilitators” [3,6,13,24,43,46] and Aδ-fibers that are “facilitated” leading to secondary hyperalgesia [3,14,16,43]. Likewise, hyperexcitability of deep dorsal horn neurons to mechanical stimulation [43] has been observed. In fact, the observed expansion of receptive field size may indirectly indicate an increase in synaptic strength, leading to increased responsiveness of dorsal horn neurons or peripheral sensitization [43,47]. Heterosynaptic facilitation of Aδ inputs might partially contribute to hyperalgesia in areas adjacent to the conditioned site [43]. Furthermore, considering secondary mechanical hyperalgesia (control site), descending mechanisms (e.g., via descending facilitation acting presynaptically on mechanosensitive nociceptors [2]) might also be involved as attenuation of dorsal horn neuronal excitability within adjacent areas by spinal transection or local anesthetic block of the rostral ventromedial medulla previously found [43,48]. These findings support the involvement of LTP in the mechanical sensitization following HFS.

Additionally, a greater magnitude of change toward mechanical hyperalgesia in conditioned and control sites, compared to adjacent conditioned sites (heterotopic sites), might suggest that HFS may enhance sensitivity to diffuse noxious inhibitory controls within the HFS-exposed hemibody, which might lead to a potential limitation of the spatial spread of secondary hyperalgesia induced by HFS [23]. Likewise, HFS-triggered activation of brainstem loops could facilitate responses to painful stimuli on the contralateral side compared to the ipsilateral side [23,49,50]. Nevertheless, the involvement of inhibitory mechanisms is not clear [51]. Thus, further studies are now needed.

### 4.2. HFS Induces Bilateral Long-Lasting Cold Hypersensitivity

A significant increase in cold sensitivity was observed at all sites after HFS in both protocols, confirming findings first reported in our previous protocol involving HFS applied to the upper limb [1]. A potential role for Aδ-fibers in cold sensitivity and hyperalgesia at conditioned and adjacent sites has been previously proposed [6,52,53]. Furthermore, disinhibited contralateral afferent information following periods of intense pain stimulation (HFS), transmitted to lumbar lamina I neurons, might explain changes observed at the contralateral site [1,8,9,54]. In fact, contralateral changes in sensitivity (QST) have been previously reported in several pain conditions [1,8,9]. Likewise, HFS may also elicit LTP of unconditioned C-fiber synapses via glial cell-mediated mechanisms [1,18].

Similar to previous studies [1,5,6,15,55], no clear significant overall changes in heat sensitivity were revealed. The absence of consistent changes in heat sensitivity suggests that possible HFS-induced plasticity is not a generalized increase in nociceptive gain but a modality-selective potentiation of the pathways recruited by the conditioning stimulus [15]. Further research is now required.

### 4.3. Influence of Temporal Summation Effect, and Demographic and Clinical Factors on Long-Lasting Hypersensitivity

Pain intensity during HFS and, hence, temporal summation effect of pain, has been associated with cold or mechanical hypersensitivity at conditioned, adjacent conditioned, and remote sites suggesting the degree of primary or secondary hyperalgesia might be related to temporal summation of pain during HFS. Nevertheless, the differences in sensitivity modalities and sensitivity sites influenced by temporal summation effect of pain between sessions suggest that different amplification mechanisms might be involved in long-lasting effects (e.g., a combination of spinal, peripheral, and descending factors [43]).

Additionally, this is the first study that showed that potential clinical factors, such as fatigue, which was a common factor influencing hypersensitivity related to cold or mechanical stimuli in both protocols (sessions), might be related to LTP-like mechanisms. In fact, previous studies found that self-reported fatigue was associated with self-reported pain intensity, pain spread, anxiety, depression, or sleep disturbance in individuals with chronic pain [56]. Nevertheless, no previous studies have investigated fatigue in HFS-induced LTP, although a possible association between attention, closely related to fatigue sharing underlying mechanisms [56], and HFS-induced LTP was previously investigated. Changes in sensitivity after HFS might be influenced by a top-down modulation operated by cognitive factors (e.g., attention), which may influence descending pain modulation system altering the spinal transmission of nociceptive inputs [21]. Top-down modulatory processes might selectively influence activity-dependent plasticity in nociceptive pathways [21]. Nevertheless, no previous studies have confirmed this hypothesis [21]. Future studies are needed to clarify these findings.

Finally, for the first time, significant age differences in mechanical pain and cold sensitivity were also observed. Although mechanical pain hypersensitivity (pinprick) was more common in older participants, younger individuals presented greater changes in mechanical pain sensitivity and cold sensitivity compared with those in older individuals. These findings might be compatible with facilitatory synaptic mechanisms resembling those described for LTP-like synaptic plasticity, whose modulation is assumed to reflect specific components of learning or memory processes [57] and might decrease as age increases. Nevertheless, previous studies have reported contradictory findings [57]. Furthermore, the present psychophysical data cannot establish whether classical spinal LTP, rather than another frequency-dependent facilitatory process, may underlie these changes. Thus, further research is required.

### 4.4. Possible Mechanisms Underlying Multimodal and Bilateral Sensory Changes After HFS

The present study highlights the bilateral expression of multimodal sensory changes after HFS. The concomitant and long-lasting changes in mechanical and cold sensitivity at conditioned, adjacent and contralateral sites indicate that HFS did not merely shift the gain of a single sensory channel but engaged convergent nociceptive circuits capable of amplifying inputs from different afferent classes [2,19]. Although the present design does not allow these mechanisms to be determined directly, this multimodal and bilateral pattern is compatible with a cascade spanning peripheral, spinal and supraspinal levels of the nociceptive system [2,18,20,43,58].

Regarding bilateral mechanical hyperalgesia, the perceptual changes at the conditioned site are compatible with homosynaptic LTP at nociceptive C-fiber synapses in the superficial dorsal horn, a N-methyl-D-aspartate (NMDA) receptor-dependent strengthening driven by glutamate release and postsynaptic calcium signaling [2,58,59]. However, whether transcutaneous HFS reliably induces homosynaptic LTP in humans remains debated [2]. The spread of hypersensitivity to adjacent and contralateral dermatomes cannot be explained by homosynaptic potentiation alone, as secondary mechanical hyperalgesia is thought to be mediated chiefly by A-fiber high-threshold mechanoreceptors and by hyperexcitability of wide-dynamic-range neurons in the deep dorsal horn. Heterosynaptic facilitation and gliogenic LTP, propagated through glial release of mediators (e.g., D-serine, tumor necrosis factor alpha), may contribute to recruiting neighboring inputs [2,18,47,58]. However, the specific contribution of C-fiber heterosynaptic LTP to secondary hyperalgesia is considered limited [2], consistent with recent evidence that “silent” C-nociceptors may contribute to, but are not essential for, the induction of secondary mechanical hyperalgesia [60].

Regarding bilateral cold hypersensitivity, cold sensitivity is largely conveyed by Aδ afferents expressing transient receptor potential channels (e.g., TRPA1). Sensitization of these cold-specific afferents, together with central amplification of their spinal synapses, offers a plausible basis for the cold hypersensitivity observed at the conditioned and adjacent sites [52,61]. Furthermore, contribution from crossed spinal sensory pathways, consistent with evidence for commissural interneuron-mediated transmission of sensory information to the contralateral side [62], might explain the presence of contralateral cold hypersensitivity.

Thus, the multimodal and bilateral sensory changes might involve spinal and/or supraspinal level.

At the spinal level, a substantial proportion of lamina I neurons receive contralateral afferent input through commissural interneurons, providing a neural substrate through which unilateral conditioning could influence nociceptive processing on the opposite side [54]. In addition, the extensive trans-segmental arborization of peripheral nociceptive afferents within the spinal cord provides a route through which conditioning at one dermatome could influence neuronal responsiveness at neighboring and remote segments [2]. Commissural convergence, together with the bilateral spread of gliogenic LTP across spinal segments [18], might partly explain the presence of contralateral cold and mechanical hypersensitivity.

Supraspinal structures are likely to contribute: the rostral ventromedial medulla (RVM) and related brainstem circuits exert bidirectional, descending control over spinal nociceptive transmission—acting in part presynaptically on mechanosensitive nociceptors—and can shift toward net facilitation after sustained noxious input, thereby amplifying dorsal horn excitability on both sides [2,43,50,63]. Cortical structures may also be involved, as the anterior cingulate cortex sends bilaterally organized, cross-callosal projections that have been shown in rodents to drive contralateral (mirror-image) hypersensitivity after a unilateral nociceptive insult [64]. Loss or imbalance of descending inhibition and engagement of diffuse descending facilitation could further extend the sensitization beyond the conditioned dermatome [43,63]. The greater magnitude of mechanical hypersensitivity observed at contralateral control sites, relative to adjacent heterotopic sites, is consistent with such a supraspinal facilitatory influence [43,50]. Consistent with these mechanisms, contralateral sensory abnormalities have repeatedly been documented in patients with unilateral neuropathic pain [8,9], supporting the clinical plausibility of the bilateral effects observed in healthy individuals. It should be emphasized that the present psychophysical findings cannot distinguish between these spinal and supraspinal contributions, which are not mutually exclusive; rather, the bilateral and multimodal pattern is most plausibly explained by their combined action on convergent nociceptive circuits [2,19,58]. Nevertheless, further research is now needed.

### 4.5. Clinical Implications

As HFS-induced LTP provides mechanistic insight into synaptic plasticity [2], this study promotes a better understanding of the possible contribution of synaptic plasticity to hyperalgesia. Likewise, understanding HFS-induced plasticity provides information related to neural sensitization and pain amplification, and, hence, potentially persistent pain. Thus, current findings suggest the potential impact of LTP-like mechanisms on chronic pain and the importance of new potential interventions focused on pain neuroplasticity mechanisms for long-term effectiveness. Additionally, new potential protocols to assess LTP-like mechanisms and, hence, neuroplasticity related to pain in any pain condition are also presented to study the potential involvement of these mechanisms in these conditions. Thus, potential predictive tools and early and comprehensive interventions for pain might be developed based on these results to improve the current management of chronic pain conditions. Nevertheless, further research is now required to support these findings.

### 4.6. Limitations

The limited sample size requires some caution to be made in the interpretation and generalization of results from the current study. Nevertheless, the sample size was higher compared with that used in previous studies [1,3,5,10,11,14,15,16,24,25]. Furthermore, other mechanisms underlying HFS-induced secondary hyperalgesia, such as reorganization of brain circuits [23], have not been studied and might be involved. Nevertheless, underlying HFS-induced secondary hyperalgesia probably reflect and highlight some of the multiple and important mechanisms that contribute to hyperalgesia observed in patients [23]. Finally, to our knowledge, this is the first study that has identified long-lasting changes in multimodal sensitivity after HFS at the adjacent test site, conditioned site and contralateral test site in two protocols with different locations, supporting the reproducibility of the current findings and promoting new comprehensive assessment and predictive tools for pain, in addition to new interventions focused on these findings. Future study of multimodal sensitization after HFS using QST protocols may provide a valuable insight into the mechanisms underlying persistent pain. Furthermore, the apparent association of age and fatigue with HFS-induced pain sensitivity highlights the need for further research examining the impact of clinical and demographic characteristics on LTP and pain sensitization in humans. Additionally, susceptibility to LTP might also be influenced by other factors, such as genetics [65,66] or physical activity [66,67]. Thus, future comprehensive research including these factors is now required.

## 5. Conclusions

The present study revealed significant bilateral long-lasting changes in cold and mechanical sensitivity following HFS applied at upper or lower limbs. The observed changes comprised a significant increase in mechanical pain sensitivity and cold sensitivity at the HFS-conditioned site and adjacent and remote test areas in both systematic upper and lower limbs dermatomal examination protocols. In addition, common long-lasting changes in cold sensitivity after HFS were dependent on age. Clinical factors, such as fatigue, were also associated with long-lasting changes in cold or mechanical sensitivity after HFS. These findings might contribute to a better understanding of physiopathological mechanisms underlying chronic pain conditions, thereby promoting the identification of potential risk factors and the development of novel predictive or diagnostic tools and new comprehensive assessments for pain. Furthermore, these results promote an innovative approach for potential new interventions for chronic pain, in addition to new management focus and prevention strategies. Future studies in chronic pain conditions are now required.

## Figures and Tables

**Figure 1:**
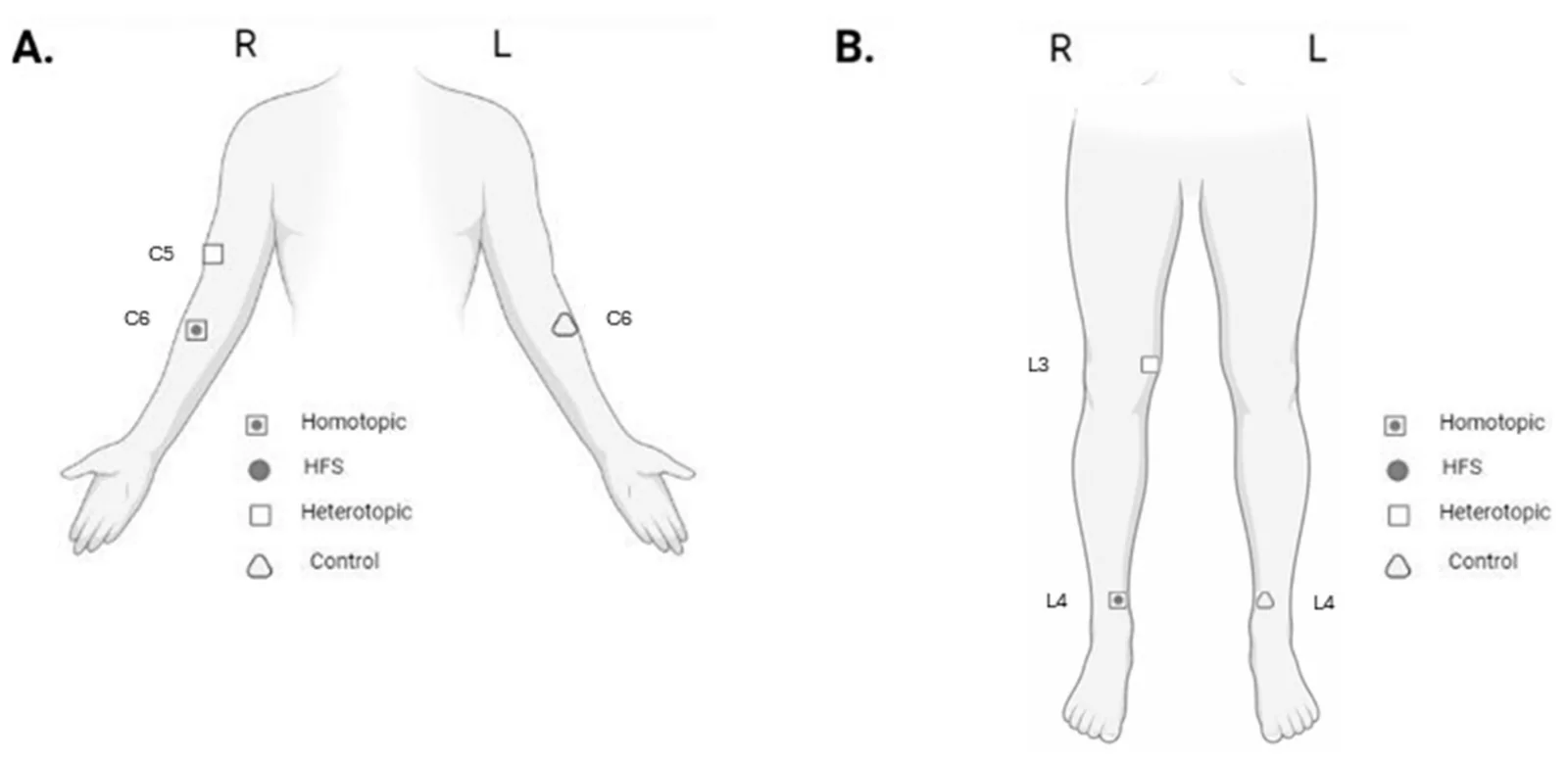
Illustrated anatomical locations (frontal plane) of the test and conditioning stimuli (HFS) for session A and session B. (**A**) Homotopic (C6 dermatome), heterotopic (C5 dermatome) and control (contralateral C6 dermatome) sites for upper limb protocol (session A). (**B**) Homotopic (L4 dermatome), heterotopic (L3 dermatome) and control (contralateral L4 dermatome) sites for lower limb protocol (session B). HFS, high-frequency stimulation; L, left (nondominant side); R, right (dominant side).

**Figure 2:**
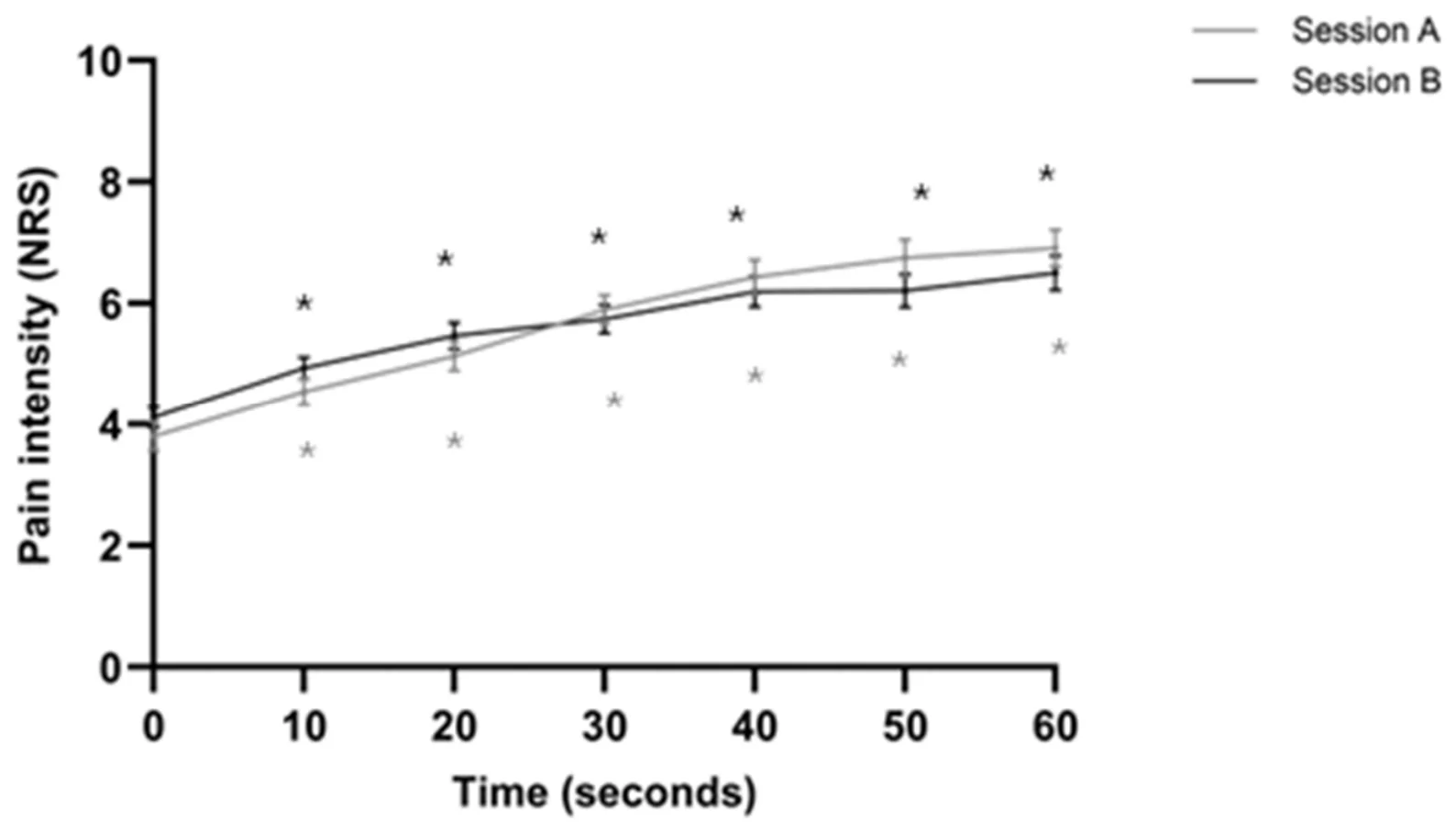
Pain ratings (NRS) perceived during HFS (60 s) in session A (upper limb protocol) and session B (lower limb protocol). * p < 0.001 (Wilcoxon matched-pairs signed-rank test). Data are presented as mean and standard error.

**Figure 3:**
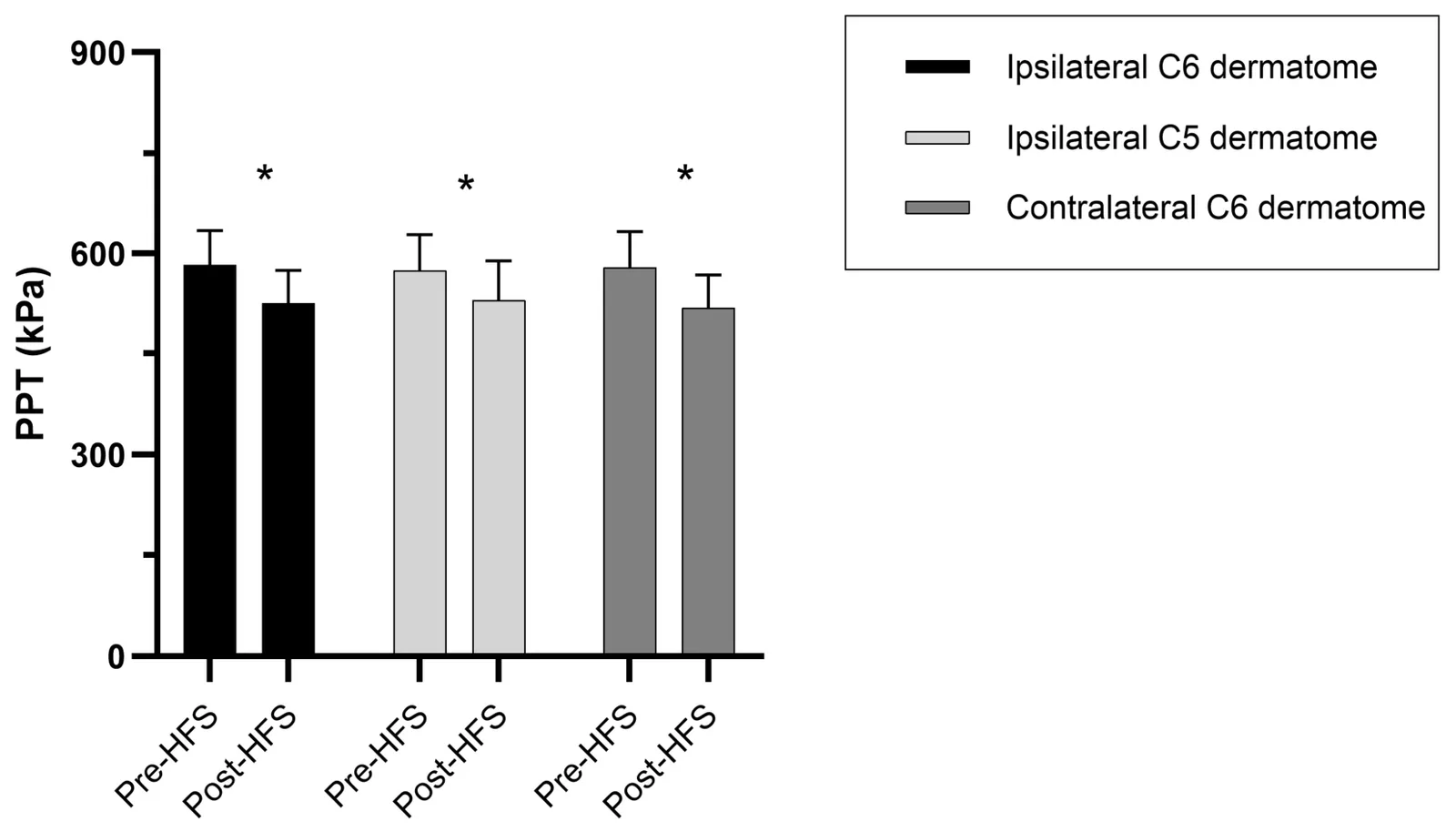
Changes in mechanical pain sensitivity (pressure pain threshold, PPT) at ipsilateral C5 and C6, and contralateral C6 dermatomes (test sites) before and after HFS (session A, upper limb protocol). * p < 0.05 (Wilcoxon matched-pairs signed-rank test). Data are presented as mean and standard error.

**Figure 4:**
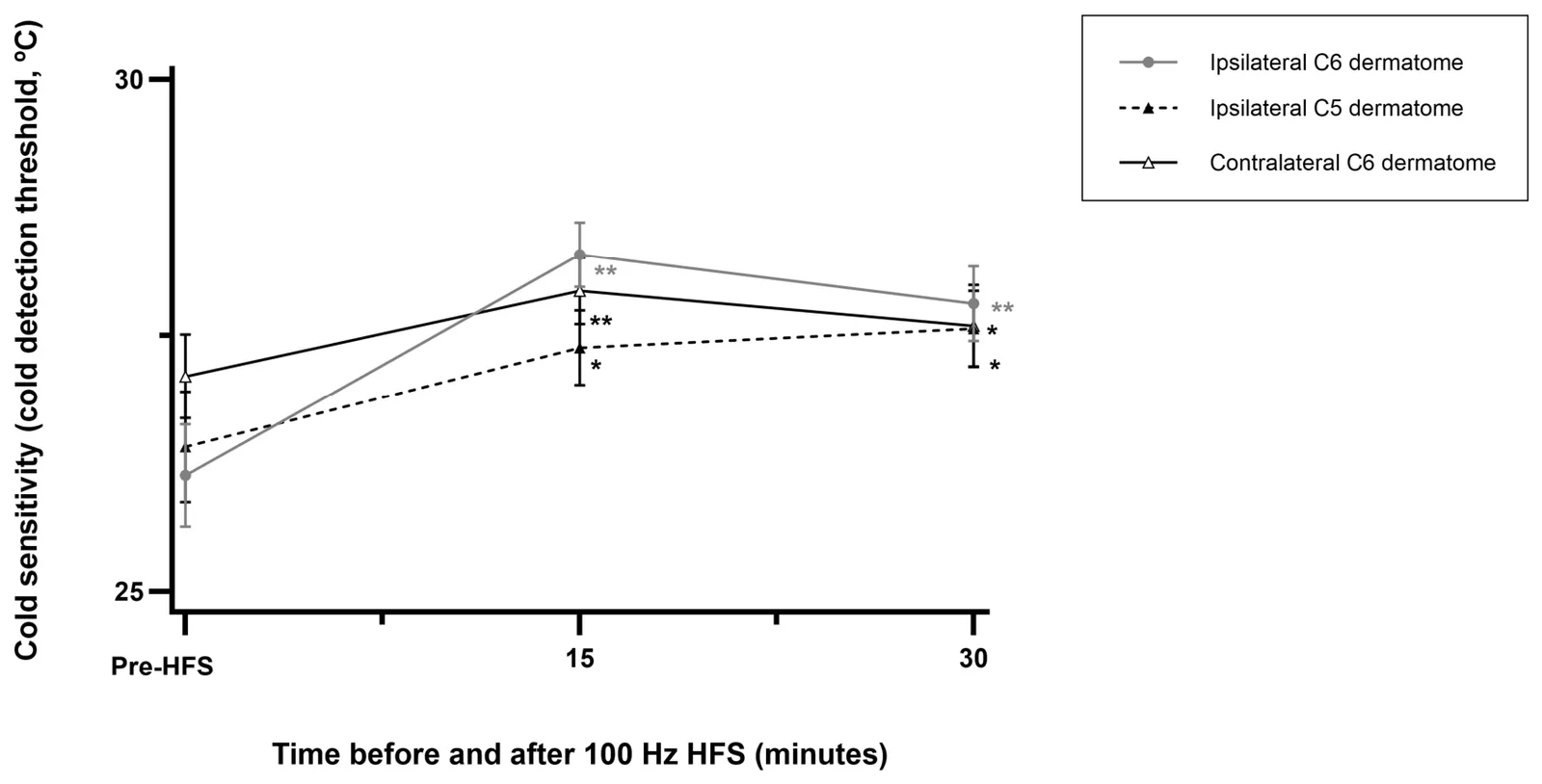
Changes in cold sensitivity at ipsilateral C5 and C6, and contralateral C6 dermatomes (test sites) before and after HFS (session A, upper limb protocol). * p < 0.05, ** p < 0.001 (Wilcoxon matched-pairs signed-rank test). Data are presented as mean and standard error.

**Figure 5:**
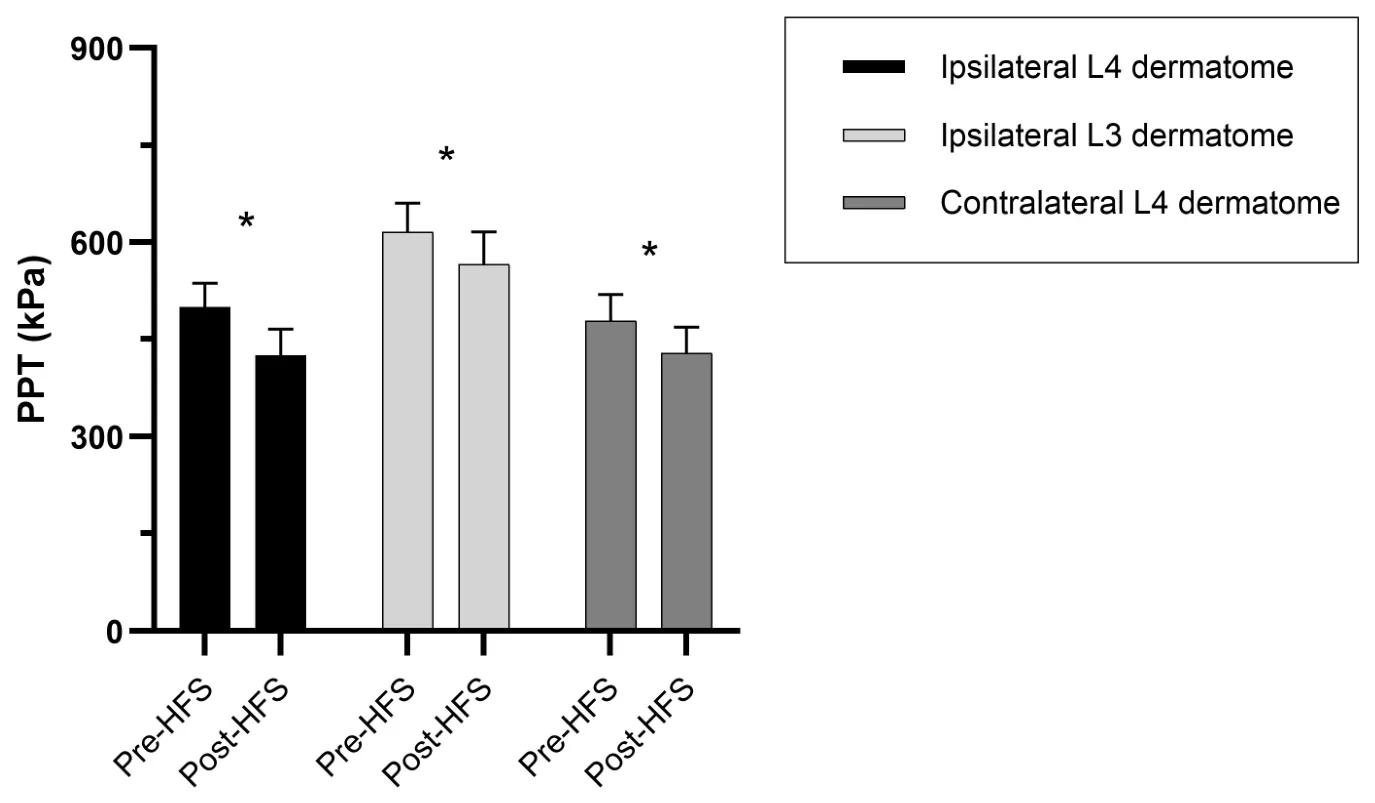
Changes in mechanical pain sensitivity (pressure pain threshold, PPT) at ipsilateral L3 and L4, and contralateral L4 dermatomes (test sites) before and after HFS (session B, lower limb protocol). * p < 0.05 (Wilcoxon matched-pairs signed-rank test). Data are presented as mean and standard error.

**Figure 6:**
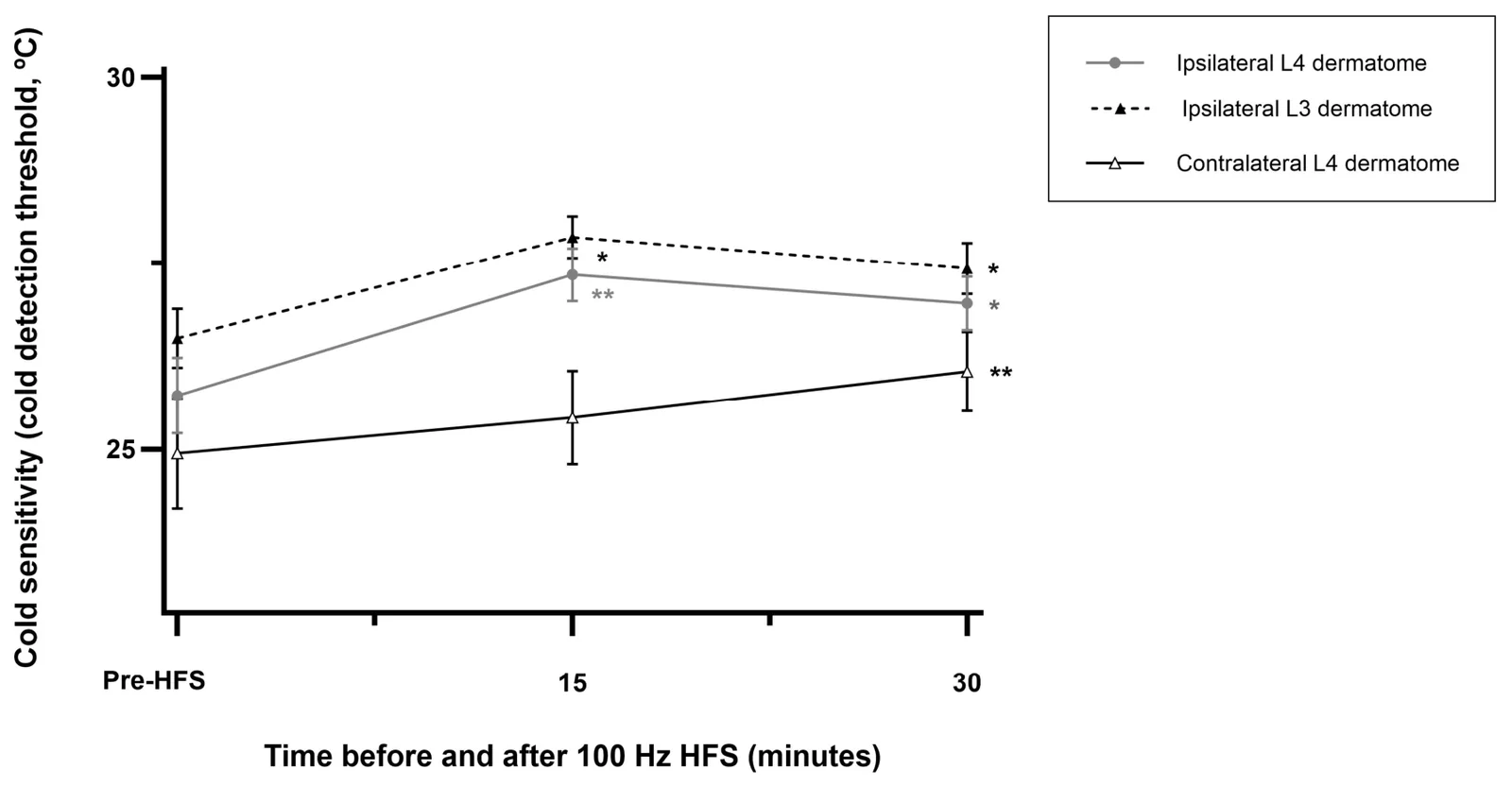
Changes in cold sensitivity at ipsilateral L3 and L4, and contralateral L4 dermatomes (test sites) before and after HFS (session B, lower limb protocol). * p < 0.05, ** p < 0.001 (Wilcoxon matched-pairs signed-rank test). Data are presented as mean and standard error.

**Table 1:** Baseline clinical and demographic characteristics of healthy participants (n = 33).

	Healthy Controls (n = 33)
Sex (male/female)	16 (48.5%)/17 (51.5%)
Age, years	29.2 (SD 8.1)
Body mass index, kg/m^2^	24.1 (SD 3.9)
EQ-5D-5L (index)	0.98 (SD 0.06)
PROMIS-29 Profile v2.1	
Physical Function T-score	57 (SD 0.0)
Anxiety T-score	48.6 (SD 7.8)
Depression T-score	43.9 (SD 6.2)
Fatigue T-score	43.4 (SD 7.0)
Sleep Disturbance T-score	46.7 (SD 8.9)
Ability to Participate in Social Roles and Activities T-score	59.1 (SD 10.7)
Pain Interference T-score	42.4 (SD 2.7)
Pain intensity (0–10)	0.1 (SD 0.4)
PASS-20 (total score)	12.3 (SD 13.8)

Data are presented as frequencies (n, %) and means (standard deviation (SD)). Abbreviations: EQ-5D-5L: the five-level version of EuroQol-5D; PASS-20: Pain Anxiety Symptoms Scale-20; PROMIS-29 Profile v2.1: Patient-Reported Outcomes Measurement Information System 29-item (PROMIS-29) profile version 2.1.

## Data Availability

The original contributions presented in this study are included in the article/Supplementary Materials. Further inquiries can be directed to the corresponding author.

## Supplementary Materials

The following supporting information can be downloaded at https://www.mdpi.com/article/10.3390/jcm15197425/s1, Table S1: Regression analysis for changes in sensitivity outcome measures related to HFS (differences pre- and post-HFS) between upper limb and lower limb protocols; Table S2: Regression analysis for changes in sensitivity outcome measures related to HFS (differences pre- and post-HFS) considering pain intensity during HFS and clinical data.

## References

[1] Escobar-Sanchez I., Rios-Leon M., Taylor J. (2023). Long-term bilateral change in pain and sensitivity to high-frequency cutaneous electrical stimulation in healthy subjects depends on stimulus modality: A dermatomal examination. Front. Med..

[2] Velasco E., van den Broeke E.N. (2026). Does long-term potentiation at spinal C-fiber synapses account for primary and secondary hyperalgesia? Insights from high-frequency stimulation studies. Pain.

[3] Klein T., Magerl W., Hopf H.C., Sandkuhler J., Treede R.D. (2004). Perceptual correlates of nociceptive long-term potentiation and long-term depression in humans. J. Neurosci..

[4] Pfau D.B., Klein T., Putzer D., Pogatzki-Zahn E.M., Treede R.D., Magerl W. (2011). Analysis of hyperalgesia time courses in humans after painful electrical high-frequency stimulation identifies a possible transition from early to late LTP-like pain plasticity. Pain.

[5] Xia W., Morch C.D., Andersen O.K. (2016). Exploration of the conditioning electrical stimulation frequencies for induction of long-term potentiation-like pain amplification in humans. Exp. Brain Res..

[6] Xia W., Morch C.D., Andersen O.K. (2016). Test-Retest Reliability of 10 Hz Conditioning Electrical Stimulation Inducing Long-Term Potentiation (LTP)-Like Pain Amplification in Humans. PLoS ONE.

[7] van den Broeke E.N., van Heck C.H., Ceelen L.A., van Rijn C.M., van Goor H., Wilder-Smith O.H. (2012). The effect of high-frequency conditioning stimulation of human skin on reported pain intensity and event-related potentials. J. Neurophysiol..

[8] Enax-Krumova E., Attal N., Bouhassira D., Freynhagen R., Gierthmuhlen J., Hansson P., Kuehler B.M., Maier C., Sachau J., Segerdahl M. (2021). Contralateral Sensory and Pain Perception Changes in Patients with Unilateral Neuropathy. Neurology.

[9] Konopka K.H., Harbers M., Houghton A., Kortekaas R., van Vliet A., Timmerman W., den Boer J.A., Struys M.M., van Wijhe M. (2012). Bilateral sensory abnormalities in patients with unilateral neuropathic pain; a quantitative sensory testing (QST) study. PLoS ONE.

[10] van den Broeke E.N., van Rijn C.M., Biurrun Manresa J.A., Andersen O.K., Arendt-Nielsen L., Wilder-Smith O.H. (2010). Neurophysiological correlates of nociceptive heterosynaptic long-term potentiation in humans. J. Neurophysiol..

[11] van den Broeke E.N., Urdi M., Mouraux A., Biurrun Manresa J.A., Torta D.M.E. (2021). High-frequency electrical stimulation of cutaneous nociceptors differentially affects pain perception elicited by homotopic and heterotopic electrical stimuli. J. Neurophysiol..

[12] van den Broeke E.N., van Heck C.H., van Rijn C.M., Wilder-Smith O.H. (2011). Neural correlates of heterotopic facilitation induced after high frequency electrical stimulation of nociceptive pathways. Mol. Pain..

[13] Henrich F., Magerl W., Klein T., Greffrath W., Treede R.D. (2015). Capsaicin-sensitive C- and A-fibre nociceptors control long-term potentiation-like pain amplification in humans. Brain.

[14] Klein T., Stahn S., Magerl W., Treede R.D. (2008). The role of heterosynaptic facilitation in long-term potentiation (LTP) of human pain sensation. Pain.

[15] Lang S., Klein T., Magerl W., Treede R.D. (2007). Modality-specific sensory changes in humans after the induction of long-term potentiation (LTP) in cutaneous nociceptive pathways. Pain.

[16] van den Broeke E.N., Vanmaele T., Mouraux A., Stouffs A., Biurrun-Manresa J., Torta D.M. (2021). Perceptual correlates of homosynaptic long-term potentiation in human nociceptive pathways: A replication study. R. Soc. Open Sci..

[17] van den Broeke E.N., Mouraux A. (2014). High-frequency electrical stimulation of the human skin induces heterotopical mechanical hyperalgesia, heat hyperalgesia, and enhanced responses to nonnociceptive vibrotactile input. J. Neurophysiol..

[18] Kronschlager M.T., Drdla-Schutting R., Gassner M., Honsek S.D., Teuchmann H.L., Sandkuhler J. (2016). Gliogenic LTP spreads widely in nociceptive pathways. Science.

[19] Sandkuhler J. (2015). Translating synaptic plasticity into sensation. Brain.

[20] Ruscheweyh R., Wilder-Smith O., Drdla R., Liu X.G., Sandkuhler J. (2011). Long-term potentiation in spinal nociceptive pathways as a novel target for pain therapy. Mol. Pain.

[21] Della Porta D., Vilz M.L., Kuzminova A., Filbrich L., Mouraux A., Legrain V. (2022). No evidence for an effect of selective spatial attention on the development of secondary hyperalgesia: A replication study. Front. Hum. Neurosci..

[22] van den Broeke E.N., Geene N., van Rijn C.M., Wilder-Smith O.H., Oosterman J. (2014). Negative expectations facilitate mechanical hyperalgesia after high-frequency electrical stimulation of human skin. Eur. J. Pain..

[23] Lebrun L., Stouffs A., Hermans E., van den Broeke E.N., Leone C., Caspani O., Truini A., Garcia-Larrea L., Treede R.D., Mouraux A. (2025). Repeated exposure to cutaneous high-frequency electrical stimulation prolongs the time course of secondary hyperalgesia. J. Neurophysiol..

[24] Hansen N., Klein T., Magerl W., Treede R.D. (2007). Psychophysical evidence for long-term potentiation of C-fiber and Adelta-fiber pathways in humans by analysis of pain descriptors. J. Neurophysiol..

[25] Klein T., Magerl W., Treede R.D. (2006). Perceptual correlate of nociceptive long-term potentiation (LTP) in humans shares the time course of early-LTP. J. Neurophysiol..

[26] World Medical Association (2013). World Medical Association Declaration of Helsinki: Ethical principles for medical research involving human subjects. JAMA.

[27] von Elm E., Altman D.G., Egger M., Pocock S.J., Gotzsche P.C., Vandenbroucke J.P., Initiative S. (2007). The Strengthening the Reporting of Observational Studies in Epidemiology (STROBE) statement: Guidelines for reporting observational studies. Lancet.

[28] Jensen M.P., Turner J.A., Romano J.M., Fisher L.D. (1999). Comparative reliability and validity of chronic pain intensity measures. Pain.

[29] Rupp R., Biering-Sorensen F., Burns S.P., Graves D.E., Guest J., Jones L., Read M.S., Rodriguez G.M., Schuld C., Tansey-Md K.E. (2021). International Standards for Neurological Classification of Spinal Cord Injury: Revised 2019. Top. Spinal Cord Inj. Rehabil..

[30] Reimer M., Forstenpointner J., Hartmann A., Otto J.C., Vollert J., Gierthmuhlen J., Klein T., Hullemann P., Baron R. (2020). Sensory bedside testing: A simple stratification approach for sensory phenotyping. Pain. Rep..

[31] Kirshblum S., Didesch M., Botticello A., Kong B., Androwis D. (2019). Patient preferences for order of the sensory portion of the International Standards for Neurological Classification of Spinal Cord Injury (ISNCSCI) examination. J. Spinal Cord Med..

[32] Vanderweeen L., Oostendorp R.A., Vaes P., Duquet W. (1996). Pressure algometry in manual therapy. Man. Ther..

[33] Nie H., Arendt-Nielsen L., Andersen H., Graven-Nielsen T. (2005). Temporal summation of pain evoked by mechanical stimulation in deep and superficial tissue. J. Pain.

[34] Chesterton L.S., Sim J., Wright C.C., Foster N.E. (2007). Interrater reliability of algometry in measuring pressure pain thresholds in healthy humans, using multiple raters. Clin. J. Pain.

[35] Lithfous S., Despres O., Pebayle T., Casadio C., Dufour A. (2020). Accurate Determination of the Cold Detection Threshold with High-Speed Cooling of the Skin. Pain. Med..

[36] Rolke R., Baron R., Maier C., Tolle T.R., Treede D.R., Beyer A., Binder A., Birbaumer N., Birklein F., Botefur I.C. (2006). Quantitative sensory testing in the German Research Network on Neuropathic Pain (DFNS): Standardized protocol and reference values. Pain.

[37] Perez C., Galvez R., Huelbes S., Insausti J., Bouhassira D., Diaz S., Rejas J. (2007). Validity and reliability of the Spanish version of the DN4 (Douleur Neuropathique 4 questions) questionnaire for differential diagnosis of pain syndromes associated to a neuropathic or somatic component. Health Qual. Life Outcomes.

[38] Lopez-Martinez A.E., Esteve R., Ruiz-Parraga G.T., Rueda-Serrano C., Serrano-Ibanez E.R., Ramirez-Maestre C. (2021). Psychometric Properties of the Spanish Version of the Pain Anxiety Symptoms Scale-20 (PASS-20-SV). Psicothema.

[39] Abrams M.P., Carleton R.N., Asmundson G.J. (2007). An exploration of the psychometric properties of the PASS-20 with a nonclinical sample. J. Pain.

[40] McCracken L.M., Dhingra L. (2002). A short version of the Pain Anxiety Symptoms Scale (PASS-20): Preliminary development and validity. Pain Res. Manag..

[41] Hernandez G., Garin O., Pardo Y., Vilagut G., Pont A., Suarez M., Neira M., Rajmil L., Gorostiza I., Ramallo-Farina Y. (2018). Validity of the EQ-5D-5L and reference norms for the Spanish population. Qual. Life Res..

[42] Rothrock N.E., Amtmann D., Cook K.F. (2020). Development and validation of an interpretive guide for PROMIS scores. J. Patient Rep. Outcomes.

[43] Patel R., Taylor J.L., Dickenson A.H., McMahon S.B., Bannister K. (2024). A back-translational study of descending interactions with the induction of hyperalgesia by high-frequency electrical stimulation in rats and humans. Pain.

[44] Biurrun Manresa J., Kaeseler Andersen O., Mouraux A., van den Broeke E.N. (2018). High frequency electrical stimulation induces a long-lasting enhancement of event-related potentials but does not change the perception elicited by intra-epidermal electrical stimuli delivered to the area of increased mechanical pinprick sensitivity. PLoS ONE.

[45] van den Broeke E.N., Lambert J., Huang G., Mouraux A. (2016). Central Sensitization of Mechanical Nociceptive Pathways Is Associated with a Long-Lasting Increase of Pinprick-Evoked Brain Potentials. Front. Hum. Neurosci..

[46] van den Broeke E.N., Mouraux A. (2014). Enhanced brain responses to C-fiber input in the area of secondary hyperalgesia induced by high-frequency electrical stimulation of the skin. J. Neurophysiol..

[47] Woolf C.J., King A.E. (1990). Dynamic alterations in the cutaneous mechanoreceptive fields of dorsal horn neurons in the rat spinal cord. J. Neurosci..

[48] Pertovaara A. (1998). A neuronal correlate of secondary hyperalgesia in the rat spinal dorsal horn is submodality selective and facilitated by supraspinal influence. Exp. Neurol..

[49] Jaggi A.S., Singh N. (2011). Role of different brain areas in peripheral nerve injury-induced neuropathic pain. Brain Res..

[50] Chen H.S., Li M.M., Shi J., Chen J. (2003). Supraspinal contribution to development of both tonic nociception and referred mirror hyperalgesia: A comparative study between formalin test and bee venom test in the rat. Anesthesiology.

[51] van den Broeke E.N., Ercken E., Maes L., Biurrun Manresa J., Torta D.M. (2026). Cold stimulation applied concurrently with high-frequency electrical stimulation does not attenuate the development of secondary pinprick hypersensitivity in humans. J. Neurophysiol..

[52] Ji G., Zhou S., Carlton S.M. (2008). Intact Adelta-fibers up-regulate transient receptor potential A1 and contribute to cold hypersensitivity in neuropathic rats. Neuroscience.

[53] Mackenzie R.A., Burke D., Skuse N.F., Lethlean A.K. (1975). Fibre function and perception during cutaneous nerve block. J. Neurol. Neurosurg. Psychiatry.

[54] Luz L.L., Lima S., Fernandes E.C., Kokai E., Gomori L., Szucs P., Safronov B.V. (2023). Contralateral Afferent Input to Lumbar Lamina I Neurons as a Neural Substrate for Mirror-Image Pain. J. Neurosci..

[55] van den Broeke E.N., Lenoir C., Mouraux A. (2016). Secondary hyperalgesia is mediated by heat-insensitive A-fibre nociceptors. J. Physiol..

[56] Holmqvist A., Berginstrom N., Lofgren M., Stalnacke B.M., Moller M.C. (2024). Fatigue and cognitive fatigability in patients with chronic pain. Scand. J. Pain..

[57] Rygvold T.W., Hatlestad-Hall C., Elvsashagen T., Moberget T., Andersson S. (2022). Long term potentiation-like neural plasticity and performance-based memory function. Neurobiol. Learn. Mem..

[58] Woolf C.J. (2011). Central sensitization: Implications for the diagnosis and treatment of pain. Pain.

[59] Sandkühler J. (2007). Understanding LTP in pain pathways. Mol. Pain.

[60] Daneffel L., Rukwied R., Schmelz M., Ruppen W., Schneider T. (2025). Preferential C-nociceptor stimulation facilitates peripheral axon reflex flare, but not secondary mechanical hyperalgesia. Front. Pain Res..

[61] McKemy D.D., Neuhausser W.M., Julius D. (2002). Identification of a cold receptor reveals a general role for TRP channels in thermosensation. Nature.

[62] Laflamme O.D., Markin S.N., Deska-Gauthier D., Banks R., Zhang Y., Danner S.M., Akay T. (2023). Distinct roles of spinal commissural interneurons in transmission of contralateral sensory information. Curr. Biol..

[63] Millan M.J. (2002). Descending control of pain. Prog. Neurobiol..

[64] Hu S.W., Zhang Q., Xia S.H., Zhao W.N., Li Q.Z., Yang J.X., An S., Ding H.L., Zhang H., Cao J.L. (2021). Contralateral Projection of Anterior Cingulate Cortex Contributes to Mirror-Image Pain. J. Neurosci..

[65] Lamb Y.N., McKay N.S., Thompson C.S., Hamm J.P., Waldie K.E., Kirk I.J. (2015). Brain-derived neurotrophic factor Val66Met polymorphism, human memory, and synaptic neuroplasticity. Wiley Interdiscip. Rev. Cogn. Sci..

[66] Spriggs M.J., Cadwallader C.J., Hamm J.P., Tippett L.J., Kirk I.J. (2017). Age-related alterations in human neocortical plasticity. Brain Res. Bull..

[67] Smallwood N., Spriggs M.J., Thompson C.S., Wu C.C., Hamm J.P., Moreau D., Kirk I.J. (2015). Influence of Physical Activity on Human Sensory Long-Term Potentiation. J. Int. Neuropsychol. Soc..

