# Supplementary material for: Bilateral Long-Lasting Changes in Multimodal Sensitivity After Cutaneous High-Frequency Electrical Stimulation: A New Comprehensive Dermatomal Examination
Source: J Clin Med. 2026 Sep 24;15(19):7425. doi: 10.3390/jcm15197425 (PMC13666166; doi:10.3390/jcm15197425)
Supplement: Supplementary file 1 [file jcm-15-07425-s001.zip › jcm-4520091-supplementary.pdf]

Table S1. Regression analysis for changes in sensitivity outcome measures related to HFS (differences pre- and post-HFS) between upper limb and lower limb protocols.

Changes in sensitivity related to HFS in upper limbs protocol *(session A)*	Changes in sensitivity related to HFS in lower limbs protocol *(session B)*
PPTs *(contralateral C6 dermatome): changes pre-HFS and post-HFS*	PPTs *(contralateral L4 dermatome): changes pre-HFS and post-HFS* *Polynomial regression analysis:* linear and quadratic model were statistically significant (R^2= 0.168, F=6.261, p=0.018; R^2= 0.184, F=3.392, p=0.047; respectively); however, the cubic model provided the best overall fit (R^2= 0.235, F=2.964, p=0.048). Although polynomial models remained significant, the increases in explained variance were modest, suggesting that the relationship exhibits slight curvature but does not substantially deviate from linearity.
Cold sensitivity *(contralateral C6 dermatome): changes pre-HFS and post-HFS (15 minutes)*	Cold sensitivity *(contralateral L4 dermatome): changes pre-HFS and post-HFS (15 minutes)* *Lineal regression analysis:* R^2=0.134, R^2 *adj*= 0.106, *b*= 1.085, *SE b*=0.495, *t*=2.191, *F*=4.799, p=0.036.
Cold sensitivity *(C6 dermatome): changes pre-HFS and post-HFS (30 minutes) **	Cold sensitivity *(L4 dermatome): changes pre-HFS and post-HFS (30 minutes) ** *Polynomial regression analysis:* linear and quadratic model were statistically significant (R^2= 0.182, F=6.905, p=0.013; R^2= 0.266, F=5.428, p=0.01; respectively); however, the cubic model provided the best overall fit (R^2= 0.289, F=3.925, p=0.018), considering that the quadratic model provided the most parsimonious fit (simplicity-parsimony). Explained variance was modest and the association exhibits mild curvature with evidence of diminishing returns.

Cold sensitivity *(C5 dermatome): changes pre-HFS and post-HFS (30 minutes)*	Cold sensitivity *(contralateral L3 dermatome): changes pre-HFS and post-HFS (30 minutes)*<br>*Polynomial regression analysis*: linear model was statistically significant (R^2= 0.155, F=5.697, p=0.023) and the quadratic model provided the best overall fit (R^2= 0.187, F=3.444, p=0.045).
Cold sensitivity *(contralateral C6 dermatome): changes pre-HFS and post-HFS (30 minutes)*	Cold sensitivity *(contralateral L4 dermatome): changes pre-HFS and post-HFS (30 minutes)*<br>*Lineal regression analysis:* R^2=0.166, R^2 *adj*= 0.139, *b*= 0.716, *SE b*=0.289, *t*=2.481, *F*=6.154, p=0.019.

Abbreviations: HFS: high-frequency stimulation; PPTs: pressure pain thresholds; SE: standard error.

*An additional preliminary analysis related to reliability (consistency) between both protocols revealed significant good consistency-reliability for this measure (intraclass correlation coefficient (ICC) = 0.602 (95% confidence interval (CI), 0.199 to 0.803), p<0.05).

Table S2. Regression analysis for changes in sensitivity outcome measures related to HFS (differences pre- and post-HFS) considering pain intensity during HFS and clinical data.

	Changes in sensitivity related to HFS
Pain intensity during HFS*	*Mechanical pain sensitivity (PPTs):*<br>*Ipsilateral C6 dermatome (session A). Polynomial regression analysis*: linear model was statistically significant *(* R^2= 0.165, F=6.119, p=0.019) and the quadratic model provided the best overall fit (R^2= 0.221, F=4.261, p=0.024).<br><br>*Cold sensitivity (15 minutes after HFS):*<br>*Ipsilateral L4 dermatome (session B):* lineal and quadratic model were statistically significant *(* R^2= 0.284, F=12.303, p=0.001; R^2= 0.326, F=7.248, p=0.003; respectively); however, the cubic model provided the best overall fit (R^2= 0.330, F=7.373, p=0.002).<br><br>*Cold sensitivity (30 minutes after HFS):*<br>*Ipsilateral C6 dermatome (session A). Polynomial regression analysis*: lineal model was statistically significant and selected as the preferred specification *(* R^2= 0.212, F=8.379, p=0.007), as higher-order terms did not yield significant improvements.<br><br>*Ipsilateral C5 dermatome (session A). Polynomial regression analysis*: lineal model was statistically significant and selected as the preferred specification *(* R^2= 0.335, F=7.563, p=0.002), as higher-order terms did not yield significant improvements.<br><br>*Contralateral C6 dermatome (session A). Polynomial regression analysis*: lineal model was statistically significant and selected as the preferred specification *(* R^2= 0.213, F=8.379, p=0.007), as higher-order terms did not yield significant improvements.

Temporal summation of pain during HFS	*Cold sensitivity (15 minutes after HFS):*<br>*Contralateral L4 dermatome (session B):* lineal model was statistically significant (R^2= 0.149, F=5.425, p=0.027) and the cubic model provided the best overall fit (R^2= 0.305, F=4.235, p=0.013).
PROMIS-29 Profile v2.1	
Physical function T-score	*Mechanical pain sensitivity (PPTs):*<br>*Contralateral L4 dermatome (session B). Polynomial regression analysis*: linear model was the unique model with statistical significance (R^2= 0.164, F=6.061, p=0.02).
Anxiety T-score	*Mechanical pain sensitivity (PPTs):*<br>*Contralateral L4 dermatome (session B). Polynomial regression analysis*: linear model was the unique model with statistical significance (R^2= 0.156, F=5.726, p=0.023).
Fatigue T-score	*Cold sensitivity (30 minutes after HFS):*<br>*Ipsilateral C6 dermatome (session A). Polynomial regression analysis*: linear model was the unique model with statistical significance (R^2= 0.225, F=6.082, p=0.022).<br><br>*Mechanical pain sensitivity (PPTs):*<br>*Contralateral L4 dermatome (session B). Polynomial regression analysis*: lineal and quadratic model were statistically significant (R^2= 0.209, F=8.201, p=0.007; R^2= 0.218, F=4.180, p=0.025; respectively); however, the cubic model provided the best overall fit (R^2= 0.247, F=3.166, p=0.039).

Sleep disturbance T-score	*Cold sensitivity (30 minutes after HFS):*<br>*Ipsilateral C5 dermatome (session A). Polynomial regression analysis*: quadratic model was statistically significant (R^2= 0.376, F=6.024, p=0.009) and the cubic model provided the best overall fit (R^2= 0.480, F=5.858, p=0.005).<br><br>*Mechanical pain sensitivity (PPTs):*<br>*Contralateral L4 dermatome (session B). Polynomial regression analysis*: lineal and quadratic model were statistically significant (R^2= 0.218, F=8.644, p=0.006; R^2= 0.222, F=4.290, p=0.023; respectively); however, the cubic model provided the best overall fit (R^2= 0.237, F=3.003, p=0.047).
EQ-5D-5L *(index)*	*Cold sensitivity (30 minutes after HFS):*<br>*Ipsilateral L3 dermatome (session B). Polynomial regression analysis*: lineal model was statistically significant (R^2= 0.182, F=6.892, p=0.013); however, the quadratic model provided the best overall fit (R^2= 0.189, F=3.499, p=0.043).

Abbreviations: EQ-5D-5L: the five-level version of EuroQol-5D; HFS: high frequency stimulation; PPTs: pressure pain thresholds; PROMIS-29 Profile v2.1: Patient-Reported Outcomes Measurement Information System 29-item (PROMIS-29) profile version 2.1.
